# Plasma Phospholipid Polyunsaturated Fatty Acid Associations with Neurocognition

**DOI:** 10.3390/nu15214542

**Published:** 2023-10-26

**Authors:** Jinjie Ling, John G. Keilp, Hanga C. Galfalvy, Vanessa N. Cardino, Alyina Ahmed, Ainsley K. Burke, Jenifer I. Fenton, J. John Mann, M. Elizabeth Sublette

**Affiliations:** 1 Department of Molecular Imaging & Neuropathology, New York State Psychiatric Institute, New York, NY 10032, USA; jinjie.ling@nyspi.columbia.edu (J.L.); john.keilp@nyspi.columbia.edu (J.G.K.); hanga.galfalvy@nyspi.columbia.edu (H.C.G.); alyina.ahmed@nyspi.columbia.edu (A.A.); ainsley.burke@nyspi.columbia.edu (A.K.B.); john.mann@nyspi.columbia.edu (J.J.M.); 2 Vagelos College of Physicians and Surgeons, Columbia University, New York, NY 10032, USA; 3 Department of Psychiatry, Columbia University, New York, NY 10032, USA; 4 Department of Biostatistics, Mailman School of Public Health, New York, NY 10032, USA; 5 Department of Food Science and Human Nutrition, Michigan State University, East Lansing, MI 48824, USA; cardinov@msu.edu (V.N.C.); imigjeni@msu.edu (J.I.F.); 6 Psychology Department, Barnard College, New York, NY 10027, USA; 7 Department of Radiology, Columbia University, New York, NY 10027, USA

**Keywords:** neurocognition, depression, suicide, omega-3 fatty acids, PUFA, executive function, attention

## Abstract

Neurocognitive deficits are implicated in major depressive disorder (MDD) and suicidal behavior, and cognitive function may be affected by blood levels of polyunsaturated fatty acids (PUFAs). Neuroprotective functions have been described for omega-3 (*n*-3) PUFAs, while omega-6 (*n*-6) PUFAs exhibit broadly opposing activities. Both classes of PUFAs are linked to MDD and suicidal behavior. However, few studies have investigated the relationships between PUFAs and neurocognitive function with respect to MDD or suicidal behavior. Among participants with MDD (*n* = 45) and healthy volunteers (HV, *n* = 30) we assessed performance on tasks of attentional capacity and executive function and its relationship to plasma phospholipid PUFA levels, expressed as a percentage of total plasma phospholipids, for eicosapentaenoic acid (EPA%), docosahexaenoic acid (DHA%), and arachidonic acid (AA%). Regression models tested the correlations between PUFA levels and task performance in three groups: MDD with a history of suicide attempt (SA, *n* = 20), MDD with no attempts (NA, *n* = 25), and HV. Interaction testing indicated a significant positive correlation of EPA% with continuous performance test scores in the NA group (*F* = 4.883, *df* = 2,72, *p* = 0.01), a measure of sustained attention. The AA% correlated negatively with performance on two executive function tasks, object alternation (*beta* = −3.97, *z*-score = −2.67, *p* = 0.008) and the Wisconsin card sort (*beta* = 0.80, *t*-score = −2.16, *df* = 69, *p* = 0.035), after adjustment for group and age, with no group effects. Our findings suggest a role for PUFA imbalance in attentional functioning and executive performance; however, no MDD-specific effect was observed.

## 1. Introduction

Neurocognitive dysfunction is implicated in the pathology of neuropsychiatric diseases, including major depressive disorder (MDD) [1,2,3] and suicidal behavior [4,5,6,7]. Potential contributors to cognitive status, in addition to symptom severity, include polyunsaturated fatty acids (PUFAs), essential components of the human diet that are vital to brain development and health [8]. In particular, the long-chain PUFAs (LC-PUFAs) arachidonic acid (AA, 20: 4 *n*-6) and docosahexaenoic acid (DHA, 22: 6 *n*-3) are highly enriched in the brain [8]. The structural properties of LC-PUFAs are determinants of function, with the omega-3 (*n*-3) LC-PUFAs DHA and eicosapentaenoic acid (EPA, 20: 5 *n*-3) generally acting in opposition to the omega-6 (*n*-6) PUFA, AA. The *n*-3 LC-PUFAs are broadly neuroprotective, acting through a variety of biological mechanisms including maintenance of membrane fluidity [9], regulation of inflammation [10], and facilitation of neurogenesis and neuronal survival [11,12]. AA, on the other hand, produces eicosanoid metabolic products that are primarily pro-inflammatory. Thus, the balance of *n*-3 to *n*-6 LC-PUFAs can powerfully affect brain functioning (reviewed in [13]).

Meta-analyses and randomized clinical trials (RCTs) interrogating the relationship between *n*-3 LC-PUFAs and neurocognitive function throughout the life cycle and in different disease contexts have produced mixed results [14,15,16].

In healthy adults, the effects of LC-PUFA supplementation on cognition are inconclusive. LC-PUFA supplementation did not enhance performance on cognitive assessments in college students and postmenopausal women [17,18], and a recent meta-analysis reported only marginal benefits on cognition from LC-PUFA supplementation in healthy individuals [14]. However, other randomized controlled trials (RCTs) have reported improvements: in the reaction time of the color-word Stroop test in young adults [19], in immediate free recall in elderly postmenopausal women [20], and in working memory in middle-aged and elderly adults [21]. In addition, lower *n*-3 LC-PUFAs correlated with worse performance on attentional tasks in young women [22].

Compared with healthy controls, patients with major depression display deficits in memory and processing tasks [23,24,25] and moderate deficits across a range of other neuropsychological tasks [26]. Memory deficits appear to persist over the long term but may depend on attentional and learning impairments [25].

Depression impairs some subtypes of effortful processing that require high cognitive capacities, regardless of the depression severity [26,27]. While moderately depressed patients tend to display memory deficits in tasks that require the creation of structure or organization, no impairment was seen in tasks that provided a structure [24]. Moreover, individuals experiencing a depressive episode displayed deficiencies in recalling words that were semantically encoded in comparison with those acoustically encoded, suggesting that depression may impair more effortful encoding [23]. Depressive symptoms have been associated with cognitive decline in older adults after controlling for demographic and clinical characteristics, including social network and other health conditions [28].

Deficits in cognitive control have a positive relationship with suicide attempts [4,5,6,7] and suicidal ideation [29] across the age spectrum [30,31,32]. Additionally, suicidal ideation is associated with greater cognitive rigidity [33], while variability in suicidal ideation is associated with poorer attention control [5]. Impaired decision-making on gambling tasks has been positively associated with ideation and attempts [34,35,36,37].

In depressed populations, the research surrounding LC-PUFAs and cognition is limited and sometimes inconclusive. Two RCTs concluded that supplementation of LC-PUFAs had negligible effects on cognitive functions and small effects on aspects of emotional cognition [38,39], whereas others reported that high EPA levels in red blood cells were associated with steeper learning curves on verbal tests [40]. A meta-analysis of *n*-3 LC-PUFA domain-specific effects on cognitive test performance in youths found no main effect of supplementation but did find a beneficial effect of EPA-rich supplements on long-term memory, working memory, and problem solving [41]. Literature regarding LC-PUFAs and cognition in suicidal populations is sparse. We located only one such study, relating *n*-3 LC-PUFA levels to cognitive reactivity [42], which found that low *n*-3 LC-PUFA levels were associated with high hopelessness and suicidality.

The current study assessed the relationship of plasma phospholipid LC-PUFA levels with performance on a battery of neuropsychiatric assessments in individuals with MDD and in healthy individuals. We conducted two assessments of attentional capacity, the continuous performance test (CPT) and the color-word Stroop test, and two assessments of executive function, the object alternation task (OAT) and the Wisconsin Card Sorting Task (WCST). Both pairs of tests have been investigated in our previous studies in relation to depression and suicidal behavior [4,6]. Given that the balance between *n*-3 and *n*-6 LC-PUFAs plays a critical physiological role in brain health and function, we hypothesized that EPA and DHA levels would correlate positively, and AA would correlate negatively, with performance on attentional and executive functioning in all participants but with the greatest impact on participants with MDD who had the highest suicide risk.

## 2. Materials and Methods

### 2.1. Sample

The present cross-sectional study was conducted at the New York State Psychiatric Institute (NYSPI, New York, NY, USA) and approved by the NYSPI Institutional Review Board. All participants provided written informed consent to participate in the study. We recruited individuals aged 18–60 years diagnosed with *Diagnostic and Statistical Manual of Mental Disorders-5* (DSM-5) MDD in a current major depressive episode (MDE; *n* = 45) and a comparison group of healthy volunteers (HV; *n* = 30), primarily through online advertisement. In the MDD group, patients were subcategorized as suicide attempters (SA; *n* = 20) or lifetime non-attempters (NA; *n* = 25), with prior suicide attempt defined as intentional self-harm with at least some intent to die.

### 2.2. Clinical Assessments

The MDD diagnoses were determined using the Structured Clinical Interview for DSM-5 (SCID-5) [43]. We excluded participants diagnosed with another major psychiatric illness, including lifetime schizophrenia, schizoaffective disorder, psychotic depression, mild substance/alcohol use disorder within the past 2 months, and moderate or severe substance/alcohol use disorder within the past 6 months. Individuals with a history of head injury were excluded if performance on the Trail Making Test indicated an existing cognitive abnormality. We did not exclude participants in the MDD group with comorbid personality disorders or participants who were taking psychotropic medications. History of suicide attempt was determined using the Columbia–Suicide Severity Rating Scale (C-SSRS) [44].

Healthy volunteers were free of psychotropic medications and without a lifetime mental health disorder diagnosis (specific phobia allowed), including a past or present history of substance/alcohol use disorder, as determined using the SCID-5. We also excluded participants who had a first-degree relative with a history of MDD, schizophrenia, schizoaffective disorder, or suicide attempt, or who had two or more first-degree relatives with a history of moderate to severe substance use disorder. Participants received comprehensive psychiatric and medical assessments, including a medical history and standard laboratory tests.

Clinical ratings were performed by Masters-level psychometricians. At the time of study entry, the 17-item Hamilton Depression Rating Scale (HDRS-17) was used to assess clinician-rated depression severity [45]. The self-reported Beck Depression Inventory (BDI) was included as an additional subjective measure of depression severity [46]. Suicidal ideation severity was evaluated using the Scale for Suicidal Ideation (SSI) [47].

### 2.3. Neurocognitive Assessments

Neuropsychological testing was performed at approximately noon for each participant, and blood sampling was performed on the same day at approximately 3 PM, after at least 3 h of fasting.

#### 2.3.1. Continuous Performance Test

To assess sustained attentional capacity, the Continuous Performance Test—Identical Pairs Version (CPT-IP) 4-digits fast condition [48] was programmed in PsyScope [49] on a Macintosh laptop computer as described previously [50]. In the CPT-IP, participants are presented with consecutive four-digit strings for 50 ms followed by 950 ms of dark time. The participant is instructed to respond if the four-digit string perfectly matches the string that directly preceded it. The main outcome measure for CPT-IP performance is the sensitivity index (d’ or d-prime) [51], which is calculated by taking the standardized difference between the hit and false alarm rates. Thus, a higher sensitivity index constitutes greater sustained attention.

#### 2.3.2. Computerized Stroop Test

To assess selective attentional capacity, we administered a computerized version of the standard Stroop test [52] in PsyScope [49] on a Macintosh computer [53]. Participants were presented with three blocks of trials with differing conditions: Word, Color, and Interference. In the Word condition, the instruction was to identify correct color names out of three different color names printed in black. In the Color condition, the instruction was to identify the color of a string of five X’s (from one of three colors). In the Interference condition, the instruction was to identify the font color while ignoring the color names when color names were presented in discordant font colors. In all conditions, the participant was instructed to respond as quickly as possible. Stimuli were presented with an interstimulus interval of 50 ms which produced a very rapid trial-to-trial pace. The main outcome measure was the interference score, calculated by computing the percentage change in median reaction time between the Color and Interference conditions. Higher Interference scores indicate reduced selective attention and attentional control.

#### 2.3.3. Object Alternation Task

The object alternation task (OAT) is a human adaptation [54] of a primate task that assesses the ability to learn and maintain a higher-order stimulus discrimination [55]. The OAT was administered via computer as described previously [56]. Briefly, the participant was simultaneously presented with a red triangle and a blue circle, with left-right positioning on the screen randomly determined. They were informed that one of the symbols was correct on any given trial, and that there was a pattern across trials to determine the correct symbol. The symbol chosen by the participant in the first trial was automatically deemed correct. Subsequently, the opposite symbol was the correct response until selected. Thereafter, the correct choice continued to alternate in this manner (without regard to the side of the display) with an intertrial interval of 500 ms. Correct responses elicited a beep; incorrect responses received a buzz. The OAT was discontinued following 12 consecutive correct identifications of alternating targets or after 180 trials if the participant was unable to achieve the test to criterion. Performance measures included the participant’s ability to achieve 12 consecutive correct responses (criterion performance) and the total number of errors.

#### 2.3.4. Wisconsin Card Sorting Task

The Wisconsin Card Sorting Task [57,58] was administered either through the standard manual version or through a computerized version on a PC laptop computer. Designed to assess prefrontal cortical dysfunction [59], the task requires the participant to sort a deck of cards containing a pseudorandom combination of four different symbols, printed in four different colors, with one to four of these symbols on each card. The participant is told that cards may be correctly sorted into one of four piles that match a model card placed above each pile. The participant is given no further instruction except that they will be given feedback for each card placement (correct or incorrect) and should use that feedback to discover any underlying rules based on features of the cards that determine correct card placement. Once the participant has made 10 sequential correct card placements, however, the rule is changed to another feature of the cards. The rule may change up to 5 times during task performance, up to the administration of 128 trials. Scores used in the analysis included criterion attainment and the number of errors made during the task. Errors can secondarily be divided into two different types: perseverative errors (continuing to respond to an older response rule that has changed) and non-perseverative errors (random inaccurate responses that are not clearly related to a prior response rule) to determine if either type is contributing more to the impaired performance.

### 2.4. Blood Sampling and Plasma Phospholipid PUFA Analysis

Blood sampling was performed at approximately the same time of day, 3 PM, for all participants, after at least 3 h of fasting. The blood samples were drawn into tubes containing ethylenediaminetetraacetic acid (EDTA), placed in an ice-water slurry, and centrifuged at low speed for 10 min under refrigerated conditions. The supernatant was allocated to cryotubes and stored at −80 °C.

Plasma glycerophospholipid fatty acids were extracted and methylated based on the methods used by Glaser, et al. [60]. Briefly, 600 μL of 5 °C methanol was added to 100 μL of each plasma sample, and the samples were vortexed. The extracted glycerophospholipids were separated with centrifugation at 2500 revolutions per minute (RPM) for 5 min at room temperature and transferred to a different tube. Then, 600 μL of 5 °C methanol was added to the plasma sample again, and the remaining extraction steps were repeated until the extracted glycerophospholipids were separated and transferred to a different tube, to yield two tubes of extracted glycerophospholipids per sample. Added to each tube was 2.917 μg of internal standard di 15:0 PC (Avanti Lipids, Alabaster, AL, USA) dissolved in 100 μL of methanol. The fatty acids (FAs) of the glycerophospholipids were transesterified into fatty acid methyl esters (FAMEs) when 25 μL of 0.5 M potassium methoxide was added to each tube. After 3 min, the reaction in each tube was neutralized with 75 μL of 9:1 (*v*/*v*) methanol:acetyl chloride. To extract the FAMEs from the rest of the sample, 600 μL of hexane was added to both tubes, and after vortexing and centrifugation using the same conditions previously mentioned, the upper hexane separations were extracted and pooled into a single tube for each sample. The combined hexane separations were dried under nitrogen, and the FAME residue was dissolved in 100 μL of isooctane (0.2% (*w*/*v*) butylated hydroxytoluene) and stored at −20 °C until gas chromatography/mass spectrometry (GC/MS) analysis. All reagents used were high-performance liquid chromatography (HPLC)-grade or higher and were purchased from Sigma-Aldrich (St. Louis, MO, USA) unless otherwise indicated.

FA quantification was performed using a Clarus 600/680 gas chromatograph/mass spectrometer (Perkin-Elmer, Waltham, MA, USA) that contained a DB-23 column (30 m length, 0.25 mm internal diameter) (Agilent, Santa Clara, CA, USA). Helium was used as the carrier gas. The gas–liquid chromatography (GC) temperature run method used was as follows: 80 °C for 5 min, ramp 4 °C/min to 240 °C, hold for 11 min. The method used selected ion monitoring to improve the sensitivity of FA detection. An external standard curve was created using GLC-248 FAME mix (Nu-Chek Prep, Elysian, MN, USA). Peak integration from the resulting chromatograms was performed using TargetLynx version 4.0.1 (Waters Corporation, Milford, MA, USA) to generate fatty acid concentrations and proportions. FAs were calculated as a percentage of total plasma glycerophospholipids, expressed as FA%. The primary species of interest for purposes of this study were DHA%, EPA%, and AA%.

### 2.5. Statistical Methods

#### 2.5.1. Statistical Software

Analysis of demographic and clinical characteristics was performed using IBM SPSS Statistics (Armonk, NY, USA; version 29.0.1.0 for Macintosh). All other statistical analyses were performed using R (version 4.3.3 for Macintosh, the R Foundation for Statistical Computing, Vienna, Austria) [61].

#### 2.5.2. Demographic and Clinical Characteristics

Clinical characteristics and demographic data were compared between the MDD and HV groups and, within the MDD group, between patients with and without a history of suicide attempt (attempters and non-attempters), using univariate analysis of variance (ANOVA) and chi-square analysis as appropriate.

#### 2.5.3. LC-PUFAs

Frequency distributions were examined for the LC-PUFAs of interest. Distributional skew was observed in EPA% and DHA%. For consistency, we performed ln-transformation on all PUFAs (EPA%, DHA%, and AA%). Following transformation, outliers (ranging from three (DHA) to five (EPA) participants) were winsorized to the nearest non-outlier value using the total sample distribution to set winsorization thresholds.

#### 2.5.4. Neurocognitive Testing

The outcome measures from the CPT-IP, Stroop, OAT, and WCST were converted to *z*-scores normalized by age, sex, and/or education using internal (see [50]) or published normative data (for WCST: [62]). Outliers (ranging from five (Stroop) to eight (WCST) participants) were identified in the total participant distribution and winsorized to the nearest non-outlier value using the total sample distribution to set winsorization thresholds.

#### 2.5.5. Statistical Analyses

Partial correlations between age-residualized LC-PUFA levels and the CPT-IP sensitivity index (d-prime), the Stroop interference measure, and the WCST error score were computed using Pearson’s coefficient. Criterion attainment was not examined in relation to the WCST because over 90% of each participant group completed the task to criterion (six categories attained) and there was an insufficient number of individuals who did not complete the task. For the OAT error score, partial correlations with age-residualized LC-PUFA levels were computed using Spearman’s coefficient. To test for differential association by group, linear regression models were generated with centered LC-PUFA as the predictor variable, group as main effect and as an interaction with the predictor, age as a covariate, and neurocognitive outcome measure as the response variable. Other pertinent covariates and cofactors were tested in all models, including sex, depression severity, suicidal ideation severity, and medication treatment status (yes/no). Non-significant covariates were removed from the final models. Because the OAT error scores demonstrated a bimodal distribution across all participant groups, reflecting two distinct sets of participants that were mostly able or mostly unable to achieve the test to criterion, a logistic regression was fit with centered LC-PUFA as the predictor variable, participant group as a main effect and through interaction, age as a covariate, and OAT attainment as the response variable. For significant group interactions, a post hoc Tukey’s test for contrasts was performed to assess the statistical significance of pair-wise group comparisons of associations and adjusted for multiple comparisons. When the interaction was not significant, it was dropped from the model.

## 3. Results

### 3.1. Demographic and Clinical Characteristics

We did not observe any significant group differences with respect to sex, race, age, or body mass index (BMI). The samples were predominantly young adult females with a BMI at the high end of the healthy range (see detailed information in Table 1, including racial breakdown of the sample). No significant differences were seen in the proportions of medication use between participants with MDD in the SA group (*n* = 12) and participants with MDD in the NA group (*n* = 10) (*c*^2^ = 1.78, *df* = 1, *p*-value = 0.182).

### 3.2. Neurocognitive Assessments

We did not observe any significant group differences with respect to performance on the CPT-IP, Stroop, OAT, or WSCT (see Table 1).

### 3.3. LC-PUFAs

We did not observe any significant group differences with respect to LC-PUFA concentrations (see Table 1).

### 3.4. LC-PUFAs and Neurocognitive Assessments

We did not detect any significant associations between DHA% and any neurocognitive outcome measures. Associations with EPA% and AA% are described below.

#### 3.4.1. Continuous Performance Test (CPT-IP)

**Figure 1:**
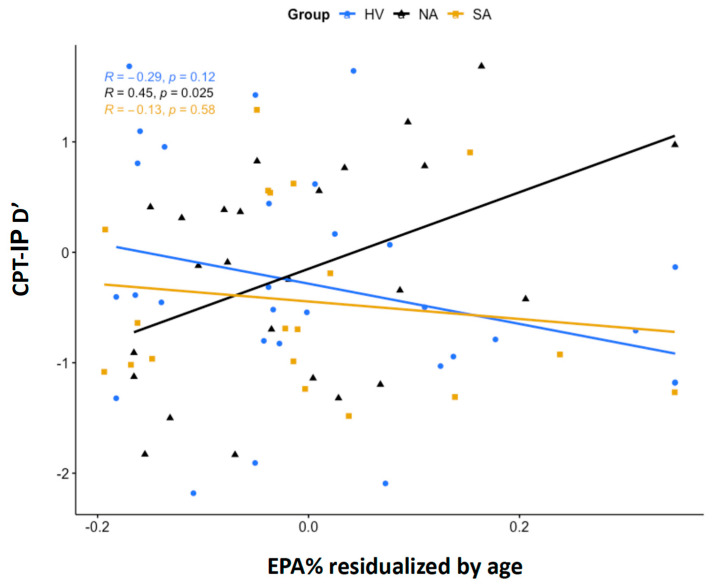
Partial correlations between eicosapentaenoic acid as a percentage of total phospholipids (EPA%) and continuous performance task performance sensitivity index (CPT-IP d’). Shown are partial correlations between age-residualized, ln-transformed EPA% and CPT-IP d’ scores in the HV, NA, and SA groups. Correlations were estimated using Pearson’s correlation coefficient, *r*.

A significant group interaction was detected in the regression model with EPA% as a predictor, age as a covariate, and CPT-IP d’ performance as the outcome (*F* = 4.883, *df* = 2,72, *p* = 0.01). A post hoc Tukey’s test for contrasts demonstrated a statistically significant difference in EPA% correlation with CPT-IP d’ between the HV and NA groups (*t* = −3.07, *df* = 67, *p* = 0.009) and a trend-level difference between the NA and SA groups (*t* = 2.24, *df* = 67, *p* = 0.072) (see Figure 1). The direct correlation of NA with d’ indicates a positive association between EPA% levels and sustained attention in this group. There were no significant main effects of sex, age, presence of medications, depression severity, or suicidal ideation.

#### 3.4.2. Computerized Stroop Test

No statistically significant relationships were seen between any LC-PUFAs and Stroop performance or reaction time (see Supplementary Tables S1–S12).

#### 3.4.3. Object Alternation Task (OAT)

There was a significant main effect in a model for AA% as a negative predictor of OAT performance (*b* = −3.97, *z*-score = −2.67, *p* = 0.008) after adjustment for participant group and age. However, the interaction with the group was not significant.

**Figure 2:**
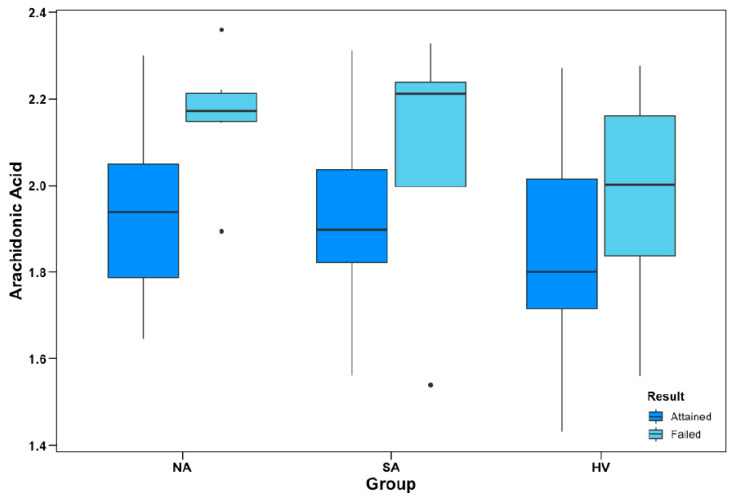
Object alternation performance and AA% levels. Group-wise levels of AA as a percentage of total plasma phospholipids (AA%) between participants who attained and failed to attain the object alternation test to criterion. AA% data are ln-transformed; apparent outliers (•) for subgroup boxplots were not considered outliers within the total sample distribution. In logistic regression models, AA% was a negative predictor of OAT performance (*p* = 0.008), covarying for group and age; however, there were no significant group interactions.

As described in the Methods section, the primary objective of the OAT is to identify and consistently respond to an alternating pattern across different trials to a criterion of 12 consecutive correct responses. Figure 2 illustrates the differences between mean AA% in participants who were able vs. unable to achieve the test to criterion across the different participant groups.

#### 3.4.4. Wisconsin Card Sort

There were no significant group interactions for any linear regression models generated with PUFA% as the predictor variable and the WCST error score as the response variable. However, there was a significant main effect of AA% as a negative predictor of WCST performance across the entire sample (*b* = 0.80, *t*-score = −2.16, *df* = 69, *p* = 0.035) after adjustment for participant group and age.

## 4. Discussion

In this study investigating the relationship between blood levels of LC-PUFAs and a selected panel of neuropsychiatric assessments, findings were partially consistent with our hypothesis that higher AA and lower *n*-3 LC-PUFA levels are associated with poorer neurocognitive performance. The two findings resulting from this study, a positive association between EPA% levels and CPT performance in MDD NA and a negative association of AA% levels with OAT and WCST performance irrespective of diagnostic group, suggest that low *n*-3 LC-PUFA and high *n*-6 LC-PUFA status may be most relevant for deficits in sustained attention and executive functioning, respectively, with no signal relating to selective attention, as assessed with the Stroop test.

The finding that EPA% levels were positively related to sustained attention in NA is consistent in directionality with our hypothesis but is unexpected in that we expected SA to be more severely affected because lower EPA levels have been linked with suicide attempts [63].

The OAT is an executive function task that requires cognitive flexibility and set maintenance and has been shown in functional imaging studies to be sensitive to dysfunction of the ventral prefrontal cortex (vPFC) [64,65], a brain region subserving cognitive control, response inhibition, and social and emotional decision-making where damage or dysfunction may result in excessive risk-taking behavior [66,67]. The WCST similarly assesses cognitive flexibility as well as working memory, which have been implicated more strongly with dorsolateral prefrontal cortex (dlPFC) function [59]. While we are aware of no published paper to date that has directly assessed the relationship between plasma phospholipid AA levels and WCST performance, one study distinguished two groups of patients with schizophrenia by high and low levels of sphingomyelin in the red blood cell (RBC) membrane composition [68]. Participants with low levels of RBC sphingomyelin had widespread perturbations in LC-PUFA species, including alterations in arachidonic acid levels, and exhibited significantly higher error rates on the WCST compared with the high-level sphingomyelin group and healthy controls.

Thus, the current study suggests that AA levels may play a role in cognitive deficits by affecting PFC function. Several previously outlined biological activities of arachidonic acid could contribute to disturbances in executive functions in the PFC, such as effects on white matter integrity [69], modulation of the endocannabinoid system [70], facilitation of long-term potentiation [71], and regulation of neurotransmission [72,73].

However, although plasma PUFAs are known to be the primary source of brain PUFAs [8], and the neurocognitive outcome measures in this study suggest a relationship between plasma phospholipid LC-PUFAs and brain functioning, relationships between peripheral and brain LC-PUFAs are complex (reviewed in [8]) and inferences regarding brain PUFA levels and cognition cannot be drawn from this study.

We did not detect any significant associations between LC-PUFAs and neurocognitive performance in the SA group. Studies investigating the relationship between suicide attempt and WCST performance have been inconsistent [4,7,74,75], and we previously reported intact object alternation in a sample of suicide attempters with a high degree of planning and non-violent methods [56]. In contrast with those findings, here we did not find a difference in the OAT error scores between the groups, which may reflect different sample characteristics or may be a consequence of smaller sample size. We note that in the current study, although performance in the patient groups was comparable to that in previous studies, healthy volunteers performed less well (see Keilp et al. [4]). Sample characteristics could have critical effects on cognition; e.g., the recency of a suicide attempt affects response inhibition and memory recognition [76]. These findings indicate that larger, better-powered studies are required to understand the relationships between LC-PUFAs and neurocognition in patients with suicidal behavior.

While the efficacy of *n*-3 LC-PUFA supplementation in MDD has been controversial [77], several studies have demonstrated improvement in depression severity following *n*-3 LC-PUFA supplementation as monotherapy or adjunctive therapy in patients with MDD [78,79,80], with EPA posited as the most crucial component for LC-PUFA supplementation [81,82,83,84,85]. Our results suggest that *n*-6 PUFA intake reduction or EPA-rich *n*-3 PUFA supplementation, which are very low-risk interventions, could improve neurocognitive deficits. However, we note that no cognitive benefits of LC-PUFA plasma levels were found specifically for MDD.

### Limitations

Our current study was limited by a small sample size. Additionally, due to the exploratory nature of the study, no correction was made for multiple comparisons. The cross-sectional study design also precludes any conclusions about causality.

## 5. Conclusions

The current study documents associations between plasma phospholipid LC-PUFA levels and executive function/sustained attention in adult patients with and without MDD. These findings may inform a therapeutic role for reduced *n*-6 PUFA intake and/or *n*-3 PUFA supplementation in neurocognitive dysfunction. However, larger prospective studies are required to test this hypothesis before recommending any dietary interventions. Ideally, future investigations would utilize fMRI and positron emission tomography with radiotracers such as [^11^C]arachidonic acid [86] or [^11^C]docosahexaenoic acid [87] to study the dynamics between peripheral and brain LC-PUFA status in relation to neurocognitive function.

## Figures and Tables

**Table 1:** Demographic and clinical characteristics of participants.

Characteristic		Total MDD (*N*%) *N* = 45		MDD SA (*N*%) *N* = 20		MDD NA (*N*%) *N* = 25		HV (*N*%) *N* = 30	Chi-Square Analysis						
									Two-Group Comparison ^1^			Three-Group Comparison ^2^			
									*c* ^2^	*df*	*p*-Value	*c* ^2^		*df*	*p*-Value
	*N*		*N*		*N*		*N*								
Sex: Male	45	24.4 (11)	20	15.0 (3)	25	32.0 (8)	30	30.0 (9)	0.903	2	0.933	4.121		4	0.390
Race: White	45	44.4 (20)	20	35.0 (7)	25	52.0 (13)	30	43.3 (13)	0.836	4	0.637	6.297		8	0.614
Asian	45	28.9 (13)	20	30.0 (6)	25	28.0 (7)	30	30.0 (9)							
Black	45	13.3 (6)	20	25.0 (5)	25	4.0 (1)	30	16.7 (5)							
More than one race	45	11.1 (5)	20	10.0 (2)	25	12.0 (3)	30	10.0 (3)							
Unknown/not reported	45	2.2 (1)	20	0.0 (0)	25	4.0 (1)	30	0.0 (0)							
**Characteristic**		**Total MDD mean (SD) *N* = 45**		**MDD SA** **mean (SD)** ***N* = 20 **		**MDD NA mean (SD)** ***N* = 25**		**HV** **mean (SD)** ***N* = 30 **	**One-way ANOVA**						
									**Two-Group Comparison ^1^**				**Three-Group Comparison ^2^**		
									***F*-Test**	***df***	***p*-Value**	***F*-Test**	***df***	***p*-Value**	**Contrast**
	***N***		***N***		***N***		***N***								
Age (yrs.)	45	28.8 (7.5)	20	28.2 (7.5)	25	29.3 (7.6)	30	26.5 (6.2)	1.898	1,73	0.172	1.083	2,72	0.344	
BMI (kg/m^2^)	44	25.9 (6.2)	20	27.0 (7.2)	24	24.9(5.1)	30	23.7 (4.0)	2.949	1,72	0.090	2.269	2,71	0.111	
HDRS (17-item)	45	18.8 (6.2)	20	19.4 (7.1)	25	18.4 (5.5)	30	0.5 (0.8)	260.305	1,73	<0.001	129.431	2,72	<0.001	SA > HV; NA > HV
BDI	45	24.8 (8.9)	20	27.8 (9.5)	25	22.5 (7.8)	30	0.9 (0.4)	208.879	1,73	<0.001	116.002	2,72	<0.001	HV < NA < SA
DHA (g/mL)	45	45.4 (15.6)	20	45.2 (17.5)	25	42.6 (14.3)	30	42.9 (18.8)	0.397	1,73	0.531	0.198	2,72	0.821	
EPA (g/mL)	45	9.9 (5.5)	20	9.8 (6.2)	25	10.0 (4.9)	30	12.7 (14.9)	1.284	1,73	0.261	0.636	2,72	0.532	
AA (g/mL)	45	125.2 (33.8)	20	120.3 (32.9)	25	129.1 (34.9)	30	114.2 (40.0)	1.632	1,73	0.206	1.632	1,73	0.206	
**Neurocognitive Assessment Scores ^3^** **Attention**		**Total MDD**		**MDD SA**		**MDD NA**		**HV**	**Analysis**						
									**Two-Group Comparison ^1^**				**Three-Group Comparison ^2^**		
									***F* or *c*^2^**		***df***	***p*-Value**	***F* or *c*^2^**	***df***	***p*-Value**
	***N***		***N***		***N***		***N***								
CPT: d-prime (mean (SD))	44	−0.3 (1.0)	19	−0.4 (0.9)	25	−0.2 (1.0)	29	−0.3 (1.0)	0.003		1,72	0.956	0.377	2,70	0.687
Stroop: Interference (%)	44	−0.5 (1.2)	20	−0.7 (1.4)	24	−0.4 (1.1)	30	−0.4 (1.2)	0.235		1,72	0.629	0.480	2,71	0.621
**Executive Function**															
OAT: Criterion Attainment (*N*%)	45	75.6 (24)	20	75.0 (15)	25	76.0 (19)	30	60.0 (18)	2.048		1	0.152	2.054	2	0.358
OAT: Errors (mean (SD))	45	−0.1 (1.1)	20	0.2 (1.2)	25	−0.4 (1.1)	30	0.1 (1.3)	0.648		1,73	0.423	1.506	2,72	0.229
WCST Criterion Attainment (*N*%)	44	88.6 (39)	20	85.0 (17)	24	91.7 (22)	30	93.3 (28)	0.495		1	0.498	1.026	2	0.599
WCST: Errors (mean (SD))	44	−0.0 (0.8)	20	0.1 (0.8)	24	−0.1 (0.8)	30	0.1 (0.7)	0.515		1,72	0.475	0.682	2,71	0.509

^1^ MDD vs. HV; ^2^ SA, NA, HV; ^3^ Neurocognitive scores are reported as age, sex, and/or education-corrected z-scores, except for criterion attainment scores, which are reported as percentages of the sample. Bold means significant difference. Abbreviations: BDI, Beck Depression Inventory; BMI, Body-Mass Index; CPT, Continuous Performance Task; HDRS, Hamilton Depression Rating Scale; MDD, major depressive disorder; NA, suicide non-attempter; OAT, Object Alternation Task; SA, suicide attempter; SD, standard deviation; WCST, Wisconsin Card Sorting Test.

## Data Availability

The data presented in this study are available on request from the corresponding author.

## Supplementary Materials

The following supporting information can be downloaded at: https://www.mdpi.com/article/10.3390/nu15214542/s1.

## References

[1] Knopman D.S., Amieva H., Petersen R.C., Chetelat G., Holtzman D.M., Hyman B.T., Nixon R.A., Jones D.T. (2021). Alzheimer disease. Nat. Rev. Dis. Primers.

[2] Pievsky M.A., McGrath R.E. (2018). The Neurocognitive Profile of Attention-Deficit/Hyperactivity Disorder: A Review of Meta-Analyses. Arch. Clin. Neuropsychol..

[3] Rock P.L., Roiser J.P., Riedel W.J., Blackwell A.D. (2014). Cognitive impairment in depression: A systematic review and meta-analysis. Psychol. Med..

[4] Keilp J.G., Gorlyn M., Russell M., Oquendo M.A., Burke A.K., Harkavy-Friedman J., Mann J.J. (2013). Neuropsychological function and suicidal behavior: Attention control, memory and executive dysfunction in suicide attempt. Psychol. Med..

[5] Herzog S., Keilp J.G., Galfalvy H., Mann J.J., Stanley B.H. (2023). Attentional control deficits and suicidal ideation variability: An ecological momentary assessment study in major depression. J. Affect. Disord..

[6] Keilp J.G., Beers S.R., Burke A.K., Melhem N.M., Oquendo M.A., Brent D.A., Mann J.J. (2014). Neuropsychological deficits in past suicide attempters with varying levels of depression severity. Psychol. Med..

[7] Keilp J.G., Sackeim H.A., Brodsky B.S., Oquendo M.A., Malone K.M., Mann J.J. (2001). Neuropsychological dysfunction in depressed suicide attempters. Am. J. Psychiatry.

[8] Bazinet R.P., Laye S. (2014). Polyunsaturated fatty acids and their metabolites in brain function and disease. Nat. Rev. Neurosci..

[9] Valentine R.C., Valentine D.L. (2004). Omega-3 fatty acids in cellular membranes: A unified concept. Prog. Lipid Res..

[10] Calder P.C. (2006). n-3 polyunsaturated fatty acids, inflammation, and inflammatory diseases. Am. J. Clin. Nutr..

[11] Dyall S.C. (2015). Long-chain omega-3 fatty acids and the brain: A review of the independent and shared effects of EPA, DPA and DHA. Front. Aging Neurosci..

[12] Bazan N.G. (2007). Omega-3 fatty acids, pro-inflammatory signaling and neuroprotection. Curr. Opin. Clin. Nutr. Metab. Care.

[13] Liu J.J., Green P., John Mann J., Rapoport S.I., Sublette M.E. (2014). Pathways of polyunsaturated fatty acid utilization: Implications for brain function in neuropsychiatric health and disease. Brain Res..

[14] Alex A., Abbott K.A., McEvoy M., Schofield P.W., Garg M.L. (2020). Long-chain omega-3 polyunsaturated fatty acids and cognitive decline in non-demented adults: A systematic review and meta-analysis. Nutr. Rev..

[15] Cooper R.E., Tye C., Kuntsi J., Vassos E., Asherson P. (2015). Omega-3 polyunsaturated fatty acid supplementation and cognition: A systematic review and meta-analysis. J. Psychopharmacol..

[16] Wood A.H.R., Chappell H.F., Zulyniak M.A. (2022). Dietary and supplemental long-chain omega-3 fatty acids as moderators of cognitive impairment and Alzheimer’s disease. Eur. J. Nutr..

[17] Karr J.E., Grindstaff T.R., Alexander J.E. (2012). Omega-3 polyunsaturated fatty acids and cognition in a college-aged population. Exp. Clin. Psychopharmacol..

[18] Ammann E.M., Pottala J.V., Harris W.S., Espeland M.A., Wallace R., Denburg N.L., Carnahan R.M., Robinson J.G. (2013). omega-3 fatty acids and domain-specific cognitive aging: Secondary analyses of data from WHISCA. Neurology.

[19] Bauer I., Hughes M., Rowsell R., Cockerell R., Pipingas A., Crewther S., Crewther D. (2014). Omega-3 supplementation improves cognition and modifies brain activation in young adults. Hum. Psychopharmacol..

[20] Strike S.C., Carlisle A., Gibson E.L., Dyall S.C. (2016). A high omega-3 fatty acid multinutrient supplement benefits cognition and mobility in older women: A randomized, double-blind, placebo-controlled pilot study. J. Gerontol. A Biol. Sci. Med. Sci..

[21] Nilsson A., Radeborg K., Salo I., Bjorck I. (2012). Effects of supplementation with n-3 polyunsaturated fatty acids on cognitive performance and cardiometabolic risk markers in healthy 51 to 72 years old subjects: A randomized controlled cross-over study. Nutr. J..

[22] Cook R.L., Parker H.M., Donges C.E., O’Dwyer N.J., Cheng H.L., Steinbeck K.S., Cox E.P., Franklin J.L., Garg M.L., O’Connor H.T. (2019). Omega-3 polyunsaturated fatty acids status and cognitive function in young women. Lipids Health Dis..

[23] Hartlage S., Alloy L.B., Vazquez C., Dykman B. (1993). Automatic and effortful processing in depression. Psychol. Bull..

[24] Hertel P.T., Rude S.S. (1991). Depressive deficits in memory: Focusing attention improves subsequent recall. J. Exp. Psychol. Gen..

[25] Hammar A., Lund A., Hugdahl K. (2003). Long-lasting cognitive impairment in unipolar major depression: A 6-month follow-up study. Psychiatry Res..

[26] Keilp J.G., Madden S.P., Gorlyn M., Burke A.K., Oquendo M.A., Mann J.J. (2018). The lack of meaningful association between depression severity measures and neurocognitive performance. J. Affect. Disord..

[27] McClintock S.M., Husain M.M., Greer T.L., Cullum C.M. (2010). Association between depression severity and neurocognitive function in major depressive disorder: A review and synthesis. Neuropsychology.

[28] Donovan N.J., Wu Q., Rentz D.M., Sperling R.A., Marshall G.A., Glymour M.M. (2017). Loneliness, depression and cognitive function in older U.S. adults. Int. J. Geriatr. Psychiatry.

[29] Nock M.K., Park J.M., Finn C.T., Deliberto T.L., Dour H.J., Banaji M.R. (2010). Measuring the suicidal mind: Implicit cognition predicts suicidal behavior. Psychol. Sci..

[30] McGirr A., Dombrovski A.Y., Butters M.A., Clark L., Szanto K. (2012). Deterministic learning and attempted suicide among older depressed individuals: Cognitive assessment using the Wisconsin Card Sorting Task. J. Psychiatr. Res..

[31] Richard-Devantoy S., Jollant F., Kefi Z., Turecki G., Olie J.P., Annweiler C., Beauchet O., Le Gall D. (2012). Deficit of cognitive inhibition in depressed elderly: A neurocognitive marker of suicidal risk. J. Affect. Disord..

[32] Richard-Devantoy S., Szanto K., Butters M.A., Kalkus J., Dombrovski A.Y. (2015). Cognitive inhibition in older high-lethality suicide attempters. Int. J. Geriatr. Psychiatry.

[33] Marzuk P.M., Hartwell N., Leon A.C., Portera L. (2005). Executive functioning in depressed patients with suicidal ideation. Acta Psychiatr. Scand..

[34] Wang S., Blazer D.G. (2015). Depression and cognition in the elderly. Annu. Rev. Clin. Psychol..

[35] Jollant F., Bellivier F., Leboyer M., Astruc B., Torres S., Verdier R., Castelnau D., Malafosse A., Courtet P. (2005). Impaired decision making in suicide attempters. Am. J. Psychiatry.

[36] Clark L., Dombrovski A.Y., Siegle G.J., Butters M.A., Shollenberger C.L., Sahakian B.J., Szanto K. (2011). Impairment in risk-sensitive decision-making in older suicide attempters with depression. Psychol. Aging.

[37] Perrain R., Dardennes R., Jollant F. (2021). Risky decision-making in suicide attempters, and the choice of a violent suicidal means: An updated meta-analysis. J. Affect. Disord..

[38] Rogers P.J., Appleton K.M., Kessler D., Peters T.J., Gunnell D., Hayward R.C., Heatherley S.V., Christian L.M., McNaughton S.A., Ness A.R. (2008). No effect of n-3 long-chain polyunsaturated fatty acid (EPA and DHA) supplementation on depressed mood and cognitive function: A randomised controlled trial. Br. J. Nutr..

[39] Antypa N., Smelt A.H., Strengholt A., Van der Does A.J. (2012). Effects of omega-3 fatty acid supplementation on mood and emotional information processing in recovered depressed individuals. J. Psychopharmacol..

[40] Emery S., Haberling I., Berger G., Baumgartner N., Strumberger M., Albermann M., Nalani K., Schmeck K., Erb S., Bachmann S. (2020). Verbal Memory Performance in Depressed Children and Adolescents: Associations with EPA but Not DHA and Depression Severity. Nutrients.

[41] Emery S., Haberling I., Berger G., Walitza S., Schmeck K., Albert T., Baumgartner N., Strumberger M., Albermann M., Drechsler R. (2020). Omega-3 and its domain-specific effects on cognitive test performance in youths: A meta-analysis. Neurosci. Biobehav. Rev..

[42] Thesing C.S., Bot M., Milaneschi Y., Giltay E.J., Penninx B. (2018). The association of omega-3 fatty acid levels with personality and cognitive reactivity. J. Psychosom. Res..

[43] First M., Williams J., Karg R., Spitzer R. (2015). Structured Clinical Interview for DSM-5—Research Version (SCID-5 for DSM-5, Research Version; SCID-5-RV).

[44] Oquendo M.A., Halberstam B., Mann J.J., First M.B. (2003). Risk factors for suicidal behavior: Utility and limitations of research instruments. Standardized Evaluation in Clinical Practice.

[45] Hamilton M. (1967). Development of a rating scale for primary depressive illness. Br. J. Soc. Clin. Psychol..

[46] Beck A.T., Ward C.H., Mendelson M., Mock J., Erbaugh J. (1961). An inventory for measuring depression. Arch. Gen. Psychiatry.

[47] Beck A.T., Kovacs M., Weissman A. (1979). Assessment of suicidal intention: The Scale for Suicide Ideation. J. Consult. Clin. Psychol..

[48] Cornblatt B.A., Risch N.J., Faris G., Friedman D., Erlenmeyer-Kimling L. (1988). The Continuous Performance Test, identical pairs version (CPT-IP): I. New findings about sustained attention in normal families. Psychiatry Res..

[49] Cohen J., Macwhinney B., Flatt M., Provost J. (1993). Psyscope—An interactive graphic system for designing and controlling experiments in the psychology laboratory using macintosh computers. Behav. Res. Methods Instrum. Comput..

[50] Keilp J.G., Gorlyn M., Oquendo M.A., Burke A.K., Mann J.J. (2008). Attention deficit in depressed suicide attempters. Psychiatry Res..

[51] Cornblatt B.A., Keilp J.G. (1994). Impaired attention, genetics, and the pathophysiology of schizophrenia. Schizophr. Bull..

[52] MacLeod C.M. (1991). Half a century of research on the Stroop effect: An integrative review. Psychol. Bull..

[53] Keilp J.G., Sackeim H.A., Mann J.J. (2005). Correlates of trait impulsiveness in performance measures and neuropsychological tests. Psychiatry Res..

[54] Freedman M., Black S., Ebert P., Binns M. (1998). Orbitofrontal function, object alternation and perseveration. Cereb. Cortex.

[55] Zald D.H., Andreotti C. (2010). Neuropsychological assessment of the orbital and ventromedial prefrontal cortex. Neuropsychologia.

[56] Keilp J.G., Wyatt G., Gorlyn M., Oquendo M.A., Burke A.K., John Mann J. (2014). Intact alternation performance in high lethality suicide attempters. Psychiatry Res..

[57] Grant D.A., Berg E.A. (1948). A Behavioral Analysis of Degree of Reinforcement and Ease of Shifting to New Responses in a Weigl-Type Card-Sorting Problem. J. Exp. Psychol..

[58] Heaton R.K. (1993). Psychological Assessment Resources (PAR) Staff. Wisconsin Card Sorting Test: Computer Version 2.

[59] Stuss D.T., Levine B., Alexander M.P., Hong J., Palumbo C., Hamer L., Murphy K.J., Izukawa D. (2000). Wisconsin Card Sorting Test performance in patients with focal frontal and posterior brain damage: Effects of lesion location and test structure on separable cognitive processes. Neuropsychologia.

[60] Glaser C., Demmelmair H., Koletzko B. (2010). High-throughput analysis of total plasma fatty acid composition with direct in situ transesterification. PLoS ONE.

[61] R Core Team (2023). R: A Language and Environment for Statistical Computing.

[62] Heaton R., Chelune G., Talley J., Kay G., Curtis G. (1993). Wisconsin Card Sorting Test Manual: Revised and Expanded.

[63] Huan M., Hamazaki K., Sun Y., Itomura M., Liu H., Kang W., Watanabe S., Terasawa K., Hamazaki T. (2004). Suicide attempt and n-3 fatty acid levels in red blood cells: A case control study in China. Biol. Psychiatry.

[64] Gold J.M., Berman K.F., Randolph C., Goldberg T.E., Weinberger D.R. (1996). PET validation of a novel prefrontal task: Delayed response alternation. Neuropsychology.

[65] Zald D.H., Curtis C., Chernitsky L.A., Pardo J.V. (2005). Frontal lobe activation during object alternation acquisition. Neuropsychology.

[66] Clark L., Manes F. (2004). Social and emotional decision-making following frontal lobe injury. Neurocase.

[67] Schmaal L., van Harmelen A.L., Chatzi V., Lippard E.T.C., Toenders Y.J., Averill L.A., Mazure C.M., Blumberg H.P. (2020). Imaging suicidal thoughts and behaviors: A comprehensive review of 2 decades of neuroimaging studies. Mol. Psychiatry.

[68] Tessier C., Sweers K., Frajerman A., Bergaoui H., Ferreri F., Delva C., Lapidus N., Lamaziere A., Roiser J.P., De Hert M. (2016). Membrane lipidomics in schizophrenia patients: A correlational study with clinical and cognitive manifestations. Transl. Psychiatry.

[69] Chhetry B.T., Hezghia A., Miller J.M., Lee S., Rubin-Falcone H., Cooper T.B., Oquendo M.A., Mann J.J., Sublette M.E. (2016). Omega-3 polyunsaturated fatty acid supplementation and white matter changes in major depression. J. Psychiatr. Res..

[70] Fagundo A.B., de la Torre R., Jimenez-Murcia S., Aguera Z., Pastor A., Casanueva F.F., Granero R., Banos R., Botella C., Del Pino-Gutierrez A. (2013). Modulation of the Endocannabinoids N-Arachidonoylethanolamine (AEA) and 2-Arachidonoylglycerol (2-AG) on Executive Functions in Humans. PLoS ONE.

[71] Williams J.H., Errington M.L., Lynch M.A., Bliss T.V.P. (1989). Arachidonic-acid induces a long-term activity-dependent enhancement of synaptic transmission in the hippocampus. Nature.

[72] Brown S.A., Morgan F., Watras J., Loew L.M. (2008). Analysis of phosphatidylinositol-4,5-bisphosphate signaling in cerebellar Purkinje spines. Biophys. J..

[73] Latham C.F., Osborne S.L., Cryle M.J., Meunier F.A. (2007). Arachidonic acid potentiates exocytosis and allows neuronal SNARE complex to interact with Munc18a. J. Neurochem..

[74] Jollant F., Lawrence N.L., Olie E., Guillaume S., Courtet P. (2011). The suicidal mind and brain: A review of neuropsychological and neuroimaging studies. World J. Biol. Psychiatry.

[75] Richard-Devantoy S., Jollant F., Deguigne F., Letourneau G. (2013). Neurocognitive markers of suicide vulnerability in the elderly: A review. Geriatr. Psychol. Neuropsychiatr. Vieil..

[76] Interian A., Myers C.E., Chesin M.S., Kline A., Hill L.S., King A.R., Miller R., Latorre M., Gara M.A., Stanley B.H. (2020). Towards the objective assessment of suicidal states: Some neurocognitive deficits may be temporally related to suicide attempt. Psychiatry Res..

[77] Lin P.Y., Mischoulon D., Freeman M.P., Matsuoka Y., Hibbeln J., Belmaker R.H., Su K.P. (2012). Are omega-3 fatty acids antidepressants or just mood-improving agents? The effect depends upon diagnosis, supplement preparation, and severity of depression. Mol. Psychiatr..

[78] Mozaffari-Khosravi H., Yassini-Ardakani M., Karamati M., Shariati-Bafghi S.E. (2013). Eicosapentaenoic acid versus docosahexaenoic acid in mild-to-moderate depression: A randomized, double-blind, placebo-controlled trial. Eur. Neuropsychopharmacol..

[79] Gertsik L., Poland R.E., Bresee C., Rapaport M.H. (2012). Omega-3 fatty acid augmentation of citalopram treatment for patients with major depressive disorder. J. Clin. Psychopharmacol..

[80] Jazayeri S., Tehrani-Doost M., Keshavarz S.A., Hosseini M., Djazayery A., Amini H., Jalali M., Peet M. (2008). Comparison of therapeutic effects of omega-3 fatty acid eicosapentaenoic acid and fluoxetine, separately and in combination, in major depressive disorder. Aust. N. Z. J. Psychiatry.

[81] Grosso G., Pajak A., Marventano S., Castellano S., Galvano F., Bucolo C., Drago F., Caraci F. (2014). Role of omega-3 fatty acids in the treatment of depressive disorders: A comprehensive meta-analysis of randomized clinical trials. PLoS ONE.

[82] Mischoulon D., Papakostas G.I., Dording C.M., Farabaugh A.H., Sonawalla S.B., Agoston A.M., Smith J., Beaumont E.C., Dahan L.E., Alpert J.E. (2009). A double-blind, randomized controlled trial of ethyl-eicosapentaenoate for major depressive disorder. J. Clin. Psychiatry.

[83] Sublette M.E., Ellis S.P., Geant A.L., Mann J.J. (2011). Meta-analysis of the effects of eicosapentaenoic acid (EPA) in clinical trials in depression. J. Clin. Psychiatry.

[84] Liao Y., Xie B., Zhang H., He Q., Guo L., Subramanieapillai M., Fan B., Lu C., McIntyre R.S. (2019). Efficacy of omega-3 PUFAs in depression: A meta-analysis. Transl. Psychiatry.

[85] Hallahan B., Ryan T., Hibbeln J.R., Murray I.T., Glynn S., Ramsden C.E., SanGiovanni J.P., Davis J.M. (2016). Efficacy of omega-3 highly unsaturated fatty acids in the treatment of depression. Br. J. Psychiatry.

[86] Zanderigo F., Kang Y., Kumar D., Nikolopoulou A., Mozley P.D., Kothari P.J., He B., Schlyer D., Rapoport S.I., Oquendo M.A. (2018). [^11^C]arachidonic acid incorporation measurement in human brain: Optimization for clinical use. Synapse.

[87] Rapoport S.I. (2013). Translational studies on regulation of brain docosahexaenoic acid (DHA) metabolism in vivo. PLEFA.

