# Supplementary material for: Plasma Phospholipid Polyunsaturated Fatty Acid Associations with Neurocognition
Source: Nutrients. 2023 Oct 26;15(21):4542. doi: 10.3390/nu15214542 (PMC10650577; doi:10.3390/nu15214542)
Supplement: Supplementary file 1 [file nutrients-15-04542-s001.zip › nutrients-2662295-supplementary.pdf]

Supplementary Tables S1-S12. Linear regression models of LC-PUFA and group interactions in neurocognitive assessment outcomes. Assessment outcomes were designated the response variable, LC-PUFA as the predictor variable, and group as a main effect and as an interaction with the predictor variable. Age was included as a covariate. Abbreviations: AA, Arachidonic Acid; CPT, Continuous Performance Task; DHA, Docosahexaenoic Acid; EPA, Eicosapentaenoic Acid; OAT, Object Alternation Task; WCST, Wisconsin Card Sort Task.

Table S1. EPA-CPT Group Interaction Model

	Df	Sum Sq	Mean Sq	F value	Pr(>F)
Age	1	3.009	3.009	3.578	0.063
Group	2	1.009	0.504	0.6	0.552
EPA	1	0.076	0.076	0.09	0.765
Group:EPA	2	8.214	4.107	4.883	0.01
Residuals	67	56.348	0.841		

Table S2. DHA-CPT Group Interaction Model

	Df	Sum Sq	Mean Sq	F value	Pr(>F)
Age	1	3.009	3.009	3.33	0.072
Group	2	1.009	0.504	0.558	0.575
DHA	1	1.813	1.813	2.006	0.161
Group:DHA	2	2.292	1.146	1.268	0.288
Residuals	67	60.533	0.903		

Table S3. AA-CPT Group Interaction Model

	Df	Sum Sq	Mean Sq	F value	Pr(>F)
Age	1	3.009	3.009	3.255	0.076
Group	2	1.009	0.504	0.546	0.582
AA	1	0.123	0.123	0.133	0.716
Group:AA	2	2.582	1.291	1.397	0.254

Table S4. EPA-Stroop Group Interaction Model

	Df	Sum Sq	Mean Sq	F value	Pr(>F)
Age	1	10.45	10.45	7.694	0.007
Group	2	1.938	0.969	0.713	0.494
EPA	1	0	0	0	0.992
Group:EPA	2	4.065	2.032	1.496	0.231
Residuals	67	90.997	1.358		

Table S5. DHA-Stroop Group Interaction Model

	Df	Sum Sq	Mean Sq	F value	Pr(>F)
Age	1	15.006	15.006	6.97	0.01
Group	2	5.811	2.906	1.35	0.266
DHA	1	2.914	2.914	1.354	0.249
Group:DHA	2	4.051	2.026	0.941	0.395
Residuals	67	144.245	2.153		

Table S6. AA-Stroop Group Interaction Model

	Df	Sum Sq	Mean Sq	F value	Pr(>F)
Age	1	15.006	15.006	7.097	0.01
Group	2	5.811	2.906	1.374	0.26
AA	1	7.375	7.375	3.488	0.066
Group:AA	2	2.167	1.084	0.513	0.601
Residuals	67	141.668	2.114		

Table S7. EPA-OAT Group Interaction Model

	Df	Deviance	Resid. Df	Resid. Dev	Pr(>Chi)
Null			74	92.461	
Age	1	9.147	73	83.314	0.002

Group	2	4.674	71	78.64	0.097
EPA	1	0.874	70	77.765	0.35
Group:EPA	2	2.216	68	75.549	0.33

Table S8. DHA-OAT Group Interaction Model

	Df	Deviance	Resid. Df	Resid. Dev	Pr(>Chi)
Null			74	92.461	
Age	1	9.147	73	83.314	0.002
Group	2	4.674	71	78.64	0.097
DHA	1	0.122	70	78.517	0.727
Group:DHA	2	0.518	68	77.999	0.772

Table S9. AA-OAT Group Interaction Model

	Df	Deviance	Resid. Df	Resid. Dev	Pr(>Chi)
Null			74	92.461	
Age	1	9.147	73	83.314	0.002
Group	2	4.674	71	78.64	0.097
AA	1	7.643	70	70.997	0.006
Group:AA	2	2.989	68	68.008	0.224

Table S10. EPA-WCST Group Interaction Model

	Df	Sum Sq	Mean Sq	F value	Pr(>F)
Age	1	5.006	5.006	9.586	0.003
Group	2	1.639	0.82	1.57	0.216
EPA	1	0.177	0.177	0.338	0.563
Group:EPA	2	0.026	0.013	0.025	0.975
Residuals	67	34.987	0.522		

Table S11. DHA-WCST Group Interaction Model

	Df	Sum Sq	Mean Sq	F value	Pr(>F)
Age	1	5.006	5.006	10.021	0.002
Group	2	1.639	0.82	1.641	0.202
DHA	1	1.275	1.275	2.552	0.115
Group:DHA	2	0.444	0.222	0.444	0.643
Residuals	67	33.471	0.5		

Table S12. AA-WCST Group Interaction Model

	Df	Sum Sq	Mean Sq	F value	Pr(>F)
Age	1	5.006	5.006	10.609	0.002
Group	2	1.639	0.82	1.737	0.184
AA	1	2.219	2.219	4.703	0.034
Group:AA	2	1.355	0.677	1.436	0.245
Residuals	67	31.615	0.472		
